# Supplementary material for: Tbps wide-field parallel optical wireless communications based on a metasurface beam splitter
Source: Nat Commun. 2024 Sep 5;15:7744. doi: 10.1038/s41467-024-52056-4 (PMC11374787; doi:10.1038/s41467-024-52056-4)
Supplement: Supplementary file 1 — Supplementary Information [file 41467_2024_52056_MOESM1_ESM.pdf]

Supplementary information for

Tbps wide-field parallel optical wireless communications based on a metasurface beam splitter

Yue Wu[1,4], Ji Chen[1,2,4]*, Yin Wang[2], Zhongyi Yuan[1], Chunyu Huang[3], Jiacheng Sun[3], Chengyi Feng[2], Muyang Li[1], Kai Qiu[3], Shining Zhu[3], Zaichen Zhang[1,2]* & Tao Li[3]*

1. National Mobile Communications Research Laboratory, School of Information Science and Engineering, Frontiers Science Center for Mobile Information Communication and Security, Quantum Information Research Center, Southeast University, Nanjing 210096, China.
2. Purple Mountain Laboratories, Nanjing 211111, China.
3. National Laboratory of Solid State Microstructures, School of Physics, Nanjing University, Nanjing, 210023, China.
4. These authors contributed equally: Yue Wu and Ji Chen.

*Corresponding authors: Ji Chen, Email: jichen@seu.edu.cn;
Zaichen Zhang, Email: zczhang@seu.edu.cn;
Tao Li, Email: taoli@nju.edu.cn.

This Supplementary information file includes:

Table S1
Notes S1 to S8
Figs. S1 to S16
Legends for Movies S1 to S3

Other Supplementary Materials for this manuscript include the following:

Movies S1 to S3

Supplementary Table S1 | Performance comparison of beam steering works

Representative works	FOV	Efficiency	Channel number	Data rate (1 channel)	Independent & Parallel	Full coverage	Method	Device Size
**Our Work**	**120°**	**40%-80%**	**144**	**200 Gbps** (28.8Tbps in total)	√	√	Metasurface	1.7mm
Adv. Mater. **34,** 2106080 (2022)<br>From State Key Laboratory of Optical Communication Technologies and Networks, China	80°	20%	14	10 Gbps	×	×	Metasurface	2mm
Nanophotonics **12,** 3511–3518 (2023).<br>From Peng Cheng Laboratory, China	20°	17.61%	9	100 Gbps (900 Gbps in total)	×	×	Metasurface	0.8mm
Science ***364,*** 1087–1090 (2019)<br>From Institute of Materials Research and Engineering, Singapore.	22°	35%	/	/	×	√	Metasurface & LC-SLM	/
Adv. Photonics Res. **4,** 2300127 (2023)<br>From State Key Laboratory of Optical Communication Technologies and Networks, China	20°	11.83%	9	10 Gbps	×	√	Metasurface & LCoS-SLM	0.8mm
J. Light. Technol. **31,** 2001–2007 (2013)<br>From University of Cambridge, UK	6°	64.5%	1	/	×	√	LC-SLM	12mm
Opt. Express **28,** 30851–30860 (2020)<br>From Huazhong University of Science and Technology, China	6°	/	8	60 Gbps	×	×	LCoS-SLM	15.4mm
Nat. Nanotechnol. **15,** 125–130 (2020).<br>From Beijing University of Technology, China	15°	40-60%	100	/	√	×	Metasurface & VCSELs	40mm
Laser Photonics Rev. **15,** 2000266 (2021)<br>From Eindhoven University of Technology, Netherlands	35°	50%	126	20 Gbps	√	√	Metasurface & AWGR	19.4× 32.63 mm
J. Light. Technol. **36,** 4486–4493 (2018)<br>From Eindhoven University of Technology, Netherlands	37.2°	/	112	80 Gbps (8.9T in total)	√	√	AWGR	50mm
IEEE Photonics Technol. Lett. **25**, 1428 (2013)<br>From Vienna University of Technology, Austria	12°	75%	1	3 Gbps	×	√	MEMS	3.6mm
IEEE Photonics Technol. Lett. **28,** 550 (2016)<br>From RIT Technologies Ltd, Israel	4°	/	8	10 Gbps	√	×	MEMS	5mm
ACS Photonics **10,** 3052–3059 (2023)<br>From Zhejiang University, China	80°	48.8%	1	10 Gbps	×	√	Metalens & translation stage	4mm× 0.6mm
Opt. Lett. **39,** 5427 (2014)<br>From Eindhoven University of Technology, Netherlands	37.2°	10-32%	1	10 Gbps	×	√	Tunable laser & Grating	/

Note: "/" means "Not measured", LC-SLM: Liquid Crystal-Spatial Light Modulator, LCoS-SLM: Liquid Crystal on Silicon-Spatial Light Modulator, VCSEL: Vertical-Cavity Surface-Emitting Laser, AWGR: Arrayed Waveguide Grating Router, MEMS: Micro-Electrical Mechanical System.

Note S1 Measurement of the divergence of beam from single-mode fiber

The fundamental mode from a step-index fiber can be theoretically approximated by Gaussian beam with the beam waist ω_0 at output end facet of the fiber. The complex field is thereby determined as detailed below:

$$E(r,z) = A_0 \frac{\omega_0}{\omega(z)} \cdot e^{-\frac{r^2}{\omega^2(z)}} \cdot e^{-i\left[k_0\left(z+\frac{r^2}{2R(z)}\right)-\tan^{-1}\left(\frac{z}{z_0}\right)\right]}. \tag{S1}$$

In Eq. (S1) r is the radial distance from the centre axis of the beam, z is the axial distance from the beam's waist, A_0 is the electric field amplitude at $(r=0, z=0)$, $\omega(z)$ is the beam radius of the fundamental mode Gaussian beam at which the field amplitudes fall to $1/e$ of their axial values at axial distance z, ω_0 is the beam waist at $z=0$, $R(z)$ is the radius of curvature at z, and z_0 is the Rayleigh length. Where

$$\begin{aligned} \omega(z) &= \omega_0\sqrt{1+\left(\frac{\lambda z}{\pi\omega_0^2}\right)^2} \\ R(z) &= z\left[1+\left(\frac{\pi\omega_0^2}{\lambda z}\right)^2\right] \\ z_0 &= \frac{\lambda}{\pi\omega_0} \end{aligned}. \tag{S2}$$

Hence, if ω_0 of the emitting beam from fiber facet is determined, the amplitude and phase of electric field at different axial distance can be simulated according to Eq. (S2). To evaluate the collimation of the beam, a beam profiler (NanoScan 2s Si/9/5) was mounted on a XYZ motorized stage and moved away from the fiber array facet with a step size of 0.5mm to measure the intensity profile of the laser, as illustrated in Fig. S1(a). The radius of the beam from a single mode fiber at different distance was figured out, accordingly ω_0 is indirectly estimated by estimating the divergence angle, where

$$\omega_0 = \frac{\lambda}{\pi\theta_{divergence}}. \tag{S3}$$

The relationship between divergence angle $\theta_{divergence}$ and $\omega(z)$ can be written as:

$$\omega(z_1) - \omega(z_2) = \tan\left(\theta_{divergence}\right)\cdot(z_1 - z_2). \tag{S4}$$

Therefore, by measuring $\omega(z)$ at different distances $z_0, z_1, \cdots, z_n$, it can be concluded that

$$\begin{bmatrix} z_1 - z_0 \\ \vdots \\ z_n - z_0 \end{bmatrix} \cdot \tan\left(\theta_{divergence}\right) = \begin{bmatrix} \omega(z_1) - \omega(z_0) \\ \vdots \\ \omega(z_n) - \omega(z_0) \end{bmatrix}. \quad \text{(S5)}$$

Thus, the least squares estimation of divergence angle $\theta_{divergence}$ is

$$\theta_{divergence} = \tan^{-1}\left((Z^T Z)^{-1} Z^T \cdot W\right). \quad \text{(S6)}$$

Accordingly, estimation of ω_0 is calculated by Eq. (S3).

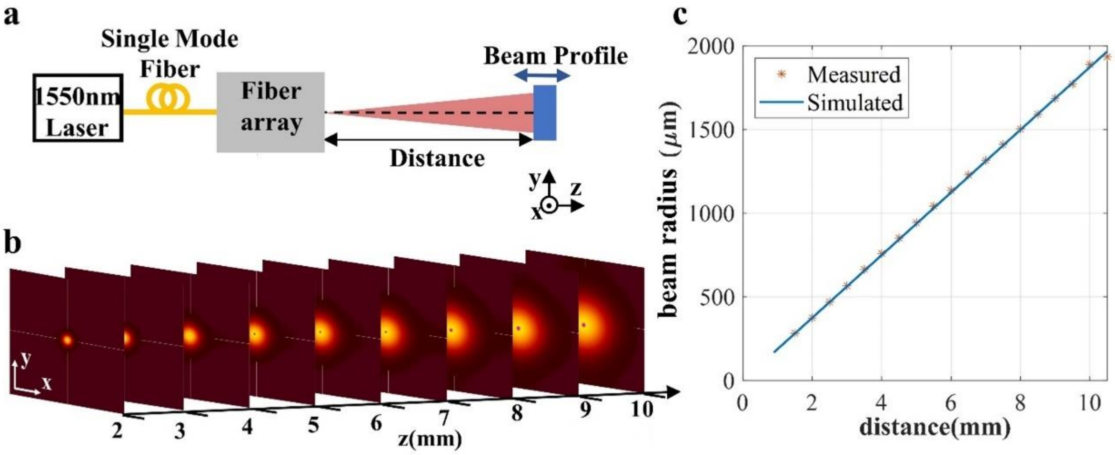

Supplementary Figure S1 | Measurement of beam divergence. (a) The experimental setup. **(b)** Recorded intensity distributions of the light beam at different distance. **(c)** Measured and estimated beam radius along axis.

As illustrated in Fig. S1(b), recorded intensity distributions of the beam away from the facet with a step of $500\mu m$ were recorded by the beam profile (NanoScan 2s, Ophir). Then, Gaussian fit was performed to the radial profile and the width at which the field amplitudes fall to $1/e$ of their axial value was extracted as a function of the distance. By performing a least squares estimation, ω_0 of beam from the fiber array was estimated to be $\omega_0 = 5.2707\mu m$. To compare the theoretical approximation with the experimental results, simulated radius of a Gaussian beam with $\omega_0 = 5.2707\mu m$ (blue solid line) and the measured radius of beam from the fiber (brown star) are plotted in Fig. S1(c) as a function of the distance *z*. As shown in Fig. S1(c), the congruence between the two curves confirms the precise measurement of the divergence. In addition, this method was also utilized to estimate ω_0 of SMF-28 Ultra FC/PC Single Mode Patch Cables (Thorlabs P1-SMF28E-FC) for verification. The beam divergences was estimated to be 5.38° and ω_0 was $5.25\mu m$, which agrees well with the mode field diameter of $10.4 \pm 0.5\mu m$ provided by the manufacturer and verifies the reliability of this method.

Note S2 Deflection ranges analysis and parameter setting of the metasurface

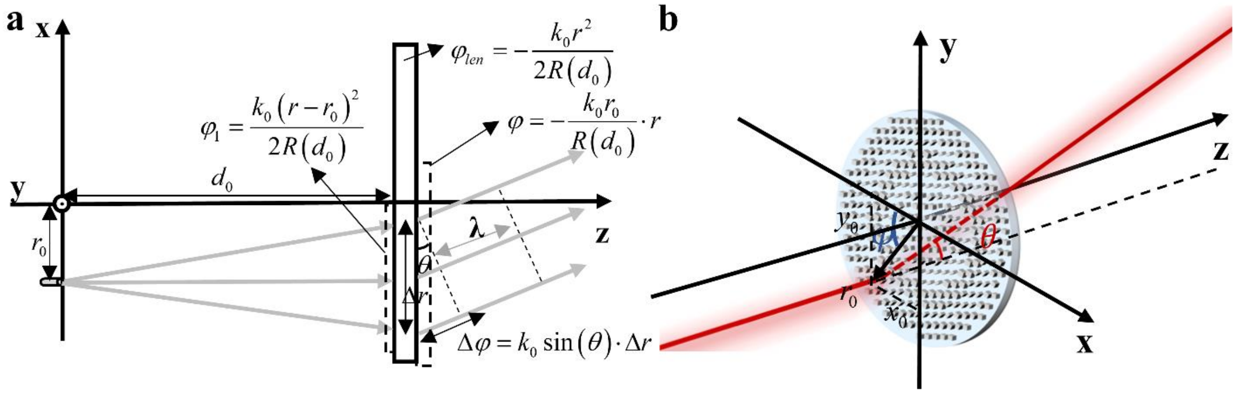

Supplementary Figure S2 | Deflection ranges of the metasurface. (a) Deflection ranges of beam from the fiber array. **(b)** Perspective view illustrating of 2D beam steering.

Phase profile of beam steering metasurface is determined as $\varphi_{lens}(r) = -k_0 r^2 / 2R(d_0)$. for fiber centred at r_0 phase distribution of the light arriving at the metasurface plane can be written as the form of quadratic phase where $\varphi_1(r) = k_0 (r - r_0)^2 / 2R(d_0)$. Consequently, the phase distribution just after the metasurface can be written as:

$$\begin{aligned}\varphi(r) &= \varphi_{lens}(r) + \varphi_1(r) = -\frac{k_0 \cdot r^2}{2R(d_0)} + \frac{k_0 \cdot (r - r_0)^2}{2R(d_0)} \\ &= -\frac{k_0 r_0 \cdot r}{R(d_0)} + \frac{k_0 \cdot r_0^{\;2}}{2R(d_0)} = -\frac{k_0 r_0}{R(d_0)} \cdot r + C\end{aligned} \quad \text{(S7)}$$

As shown in Fig. S2(a) the gradient of phase $-k_0 r_0 / R(d_0)$ can be expressed as the phase gradient of oblique projection with deflection angle of $\theta = \sin^{-1}(-r_0 / R(d_0))$. To implement 2D deflection angles, the linear coordinate is replaced by radial coordinate as shown in Fig. S2(b). The beam is deflected to an outgoing general angle θ lying within the orthogonal plane to the metasurface, which exhibits symmetrical behaviour for any elevation angle φ. The azimuthal θ is only related to the radial distance $|r| = \sqrt{x_0^{\;2} + y_0^{\;2}}$, thus we obtain:

$$\begin{cases} \theta = \sin^{-1}\left(-\frac{r}{R(d_0)}\right) \\ \varphi = \tan^{-1}\left(\frac{y_0}{x_0}\right) \end{cases} . \quad \text{(S8)}$$

According to Eq. (S8), the deflection range can be calculated as:

$$\theta_{range} = \left| \sin^{-1}\left(\frac{r_{max}}{R(d_0)}\right) - \sin^{-1}\left(\frac{r_{min}}{R(d_0)}\right) \right|. \tag{S9}$$

Where $|r| = \sqrt{x_0{}^2 + y_0{}^2}$ is the radial distance from the beam centre to the metasurface centre, d_0 is the distance between fiber array facet and the metasurface, $R(d_0) = d_0 \cdot \left[1 + \left(\pi\omega_0^2/\lambda d_0\right)^2\right]$ is the curvature radius of Gaussian beam at distance d_0.

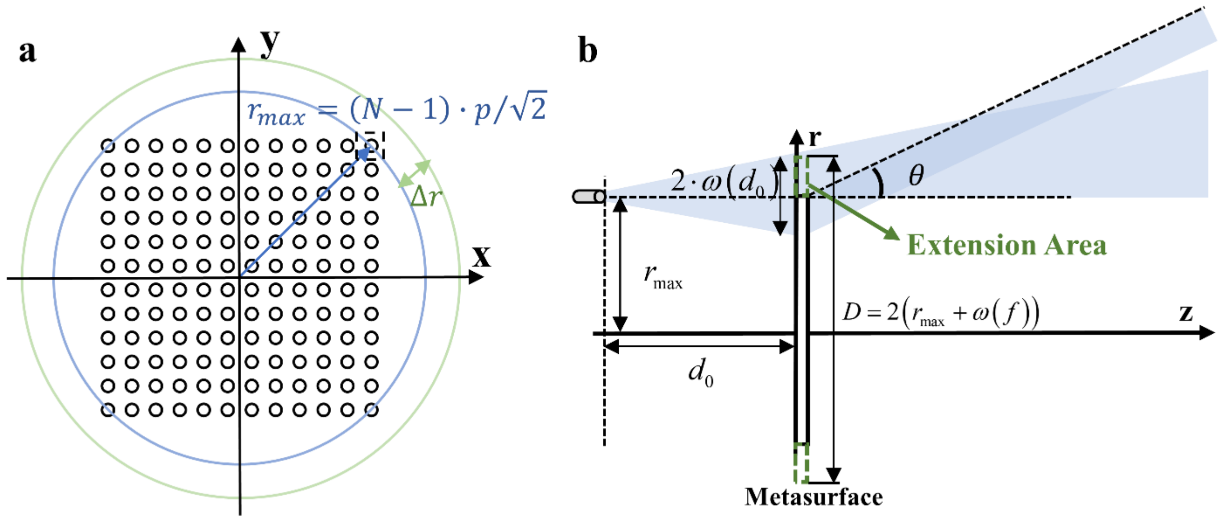

Supplementary Figure S3 | Effective aperture size of the metasurface. (a) Top view of the metasurface and fiber array. **(b)** side views of the metasurface with real diameter.

For the fiber array contains N fibers with intervals of p, the radial distance of fiber to the centre of metasurface satisfies $-(N-1)\cdot p/\sqrt{2} \le r \le (N-1)\cdot p/\sqrt{2}$, as shown in Fig. S3(a). It is observed that the distance d_0 is determined once the five parameters λ, ω_0, N, p and θ_{range} are determined. In this paper, a fiber array comprising 12×12 fiber with intervals of $100\mu m$ is utilized as the emitter. The beam waist at the end facet of the fiber in the array ω_0 is approximately 5.27μm at working wavelength $\lambda = 1550nm$ (refer to Section 1 of the Supporting Information). θ_{range} of the metasurface is determined as $120°$ and the maximum deflection angle is determined as $60°$, thus $R(d_0)$ is determined as $r_{max}/\sin(60°) = 898.15\mu$m. Accordingly, d_0 can be calculated according to the value of λ and ω_0 as $900\mu m$.

$$R(d_0) = d_0\sqrt{1 + \left(\frac{\pi\omega_0^2}{\lambda d_0}\right)^2} \Rightarrow R^2(d_0) = d_0{}^2 + \left(\frac{\pi\omega_0^2}{\lambda}\right)^2 \Rightarrow d_0 = \sqrt{R^2(d_0) - \left(\frac{\pi\omega_0^2}{\lambda}\right)^2}. \tag{S10}$$

The dimension of the Gaussian beam spot at the metasurface plane and the physical size of the actual metasurface are also taken into consideration. In order to encompass all the light emitted from the fiber array, the diameter of the metasurface donates as D must be no less than $2 \cdot r_{\max}$. However, as depicted in Fig. S3(b), for the metasurface with diameter $D = 2 \cdot r_{\max}$, only a portion of the light irradiates the effective region of the metasurface. Light rays that do not impinge upon the effective region of the metasurface are not collimated and steered by the metasurface, instead propagating in their original trajectory and exhibiting divergence. Therefore, to ensure that a substantial amount of the emitted light's energy is captured within the effective region of the metasurface, the diameter of the metasurface is expanded to $D = 2 \cdot \left(r_{\max} + \omega\left(d_0 \right) \right)$. In this paper the diameter of the metasurface is determined to be 1.725mm.

Note S3 Influence of metasurface chromatic aberration on deflection angle

The electric field of light emitted from a fiber port can be theoretically approximated by a Gaussian beam model as shown in Eq. (1) in the main text. The phase profile just before the metasurface placed in front of the fiber with a distance of d0 would be simplified as a quadratic phase form shown as: $\varphi_1(r)=kr^2/2R\left(d_0\right)+C$. Where $k=2\pi/\lambda$ is the wave number of the light with wavelength of λ, $R\left(d_0,\lambda\right)=d_0\left[1+\left(\pi\omega_0^2/\lambda d_0\right)^2\right]$ is the function of d_0 and λ. The phase distribution of the metasurface designed for λ_0=*1550nm* can be expressed as $\varphi_{lens}(r)=-k_0r^2/2R\left(d_0\left(\lambda_0\right)\right)$.

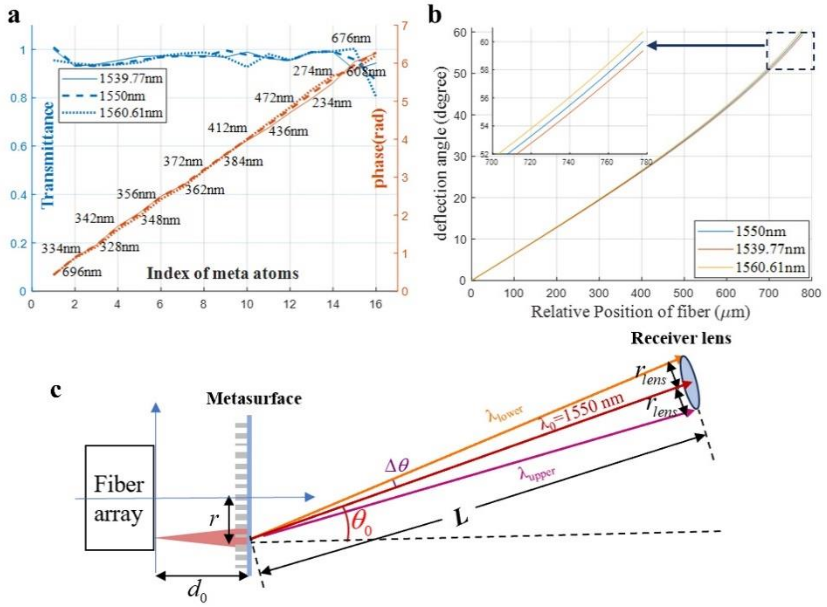

Supplementary Figure S4 | Influence of metasurface chromatic aberration on deflection angle. (a) Simulated transmittance and phase shift of meta atoms of three wavelengths. **(b)** Deflection angle as a function of illuminating position r, at wavelengths of 1550 nm, 1539.77 nm, and 1560.61 nm, respectively. **(c)** Geometric relationship of deflection angle changes at different wavelengths. It should be noted that, for clarity of illustration, some length proportions in the figure are not to scale.

Optical response of the 16 nano-posts with different diameters were identified at the wavelength of 1539.77nm (solid line), 1550.12nm (dashed line) and 1560.61nm (dotted line) as shown in Fig. S4(a). The 16 nano-posts exhibits equivalent responsiveness to light at 1539.77nm and 1560.61nm as observed at 1550nm, which infers the consistent phase delay of the metasurface across different wavelengths within the bandwidth of the WDM OWC system. Thus, when the divergent beam passes through the metasurface at position r_0, the phase distribution just after the metasurface can be written as:

$$
\begin{aligned}
\varphi(r) &= \varphi_{lens}(r)+\varphi_1(r) = -\frac{k_0\cdot r^2}{2R(d_0,\lambda_0)}+\frac{k\cdot(r-r_0)^2}{2R(d_0,\lambda)} \\
&= \frac{\left(k\cdot R(d_0,\lambda_0)-k_0\cdot R(d_0,\lambda)\right)\cdot r^2}{2R(d_0,\lambda_0)\cdot R(d_0,\lambda)}-\frac{k\cdot r_0\cdot r}{R(d_0,\lambda)}+\frac{k\cdot {r_0}^2}{2R(d_0,\lambda)} . \\
&= \frac{\left(k\cdot R(d_0,\lambda_0)-k_0\cdot R(d_0,\lambda)\right)\cdot r^2}{2R(d_0,\lambda_0)\cdot R(d_0,\lambda)}-\frac{k\cdot r_0}{R(d_0,\lambda)}\cdot r+C.
\end{aligned}
\tag{S11}
$$

Eq. (S11) consisted of a first-order term that influences the deflection angle of the emitted light, a second-order term that influences the divergence angle of the emitted light, and a constant term. Submitting $R(d_0,\lambda)=d_0\left[1+\left(\pi\omega_0^2/\lambda d_0\right)^2\right]$into the second-order term, the second-order term of Eq. (S11) can be written as:

$$
\begin{aligned}
&\frac{\left(k\cdot R(d_0,\lambda_0)-k_0\cdot R(d_0,\lambda)\right)\cdot r^2}{2R(d_0,\lambda_0)\cdot R(d_0,\lambda)} \\
&= \frac{r^2}{2}\cdot\frac{k\cdot\left(\pi\omega_0^2/\lambda_0 d_0\right)^2-k_0\cdot\left(\pi\omega_0^2/\lambda d_0\right)^2+k-k_0}{1+\left(\pi\omega_0^2/\lambda_0 d_0\right)^2+\left(\pi\omega_0^2/\lambda d_0\right)^2+\left(\pi\omega_0^2/\lambda d_0\right)^2\cdot\left(\pi\omega_0^2/\lambda_0 d_0\right)^2} .
\end{aligned}
\tag{S12}
$$

Since $\pi\omega_0^2/\lambda d_0$ <<1, $k-k_0=2\pi\cdot(\lambda_0-\lambda)/\lambda_0\cdot\lambda$ <<1, the second-order term of Eq. (S11) approaches zero for all the wavelengths used in the WDM OWC systems. It can be inferred that, the metasurface exhibits uniform capability of transforming a divergent light beam into a precisely collimated one within the bandwidth of the WDM OWC systems. The gradient of the phase of metasurface can be expressed as the phase gradient of oblique projection with deflection angle determined by the generalized Snell's law, which would be expressed as:

$$
\begin{aligned}
\sin\theta(r_0) &= \frac{1}{k}\frac{\partial}{\partial r}\left(\varphi(r)\right)\Big|_{r=r_0} = \frac{1}{k}\cdot\left(-\frac{r_0\cdot k}{R(d_0,\lambda)}+\frac{\left(k\cdot R(d_0,\lambda_0)-k_0\cdot R(d_0,\lambda)\right)\cdot r_0}{R(d_0,\lambda_0)\cdot R(d_0,\lambda)}\right) \\
&= \frac{1}{k}\cdot\left(-\frac{k_0\cdot r_0}{R(d_0,\lambda_0)}\right) = -\frac{\lambda}{\lambda_0}\cdot\frac{r_0}{d_0\left[1+\left(\pi\omega_0^2/\lambda d_0\right)^2\right]} .
\end{aligned}
\tag{S13}
$$

The deflection angle of three wavelengths at different relative position between the fiber center and metasurface are plotted in Fig. S4(b). Due to the small wavelength deviation from the center wavelength of 1550 nm, the deflection angle changes are also very small.

However, when the communication distance is relatively long, the deflection angle changes cannot be ignored. The geometric relationship of the beam deflection angle, communication distance, and the receiver lens size is illustrated in Fig. S4(c). For laser with the wavelength of λ, the deviation in the deflection angle results in a shift of the light spot at the receiving plane located at a distance of L as $r_{shift}=\tan\left(\theta(r,\lambda)-\theta_0(r,\lambda_0)\right)\cdot L$. To ensure that sufficient energy can be

coupled through the receiver collimator into the fiber, the centers of light spots at different wavelengths must fall within the effective area of the coupling lens. To be precise, the offset of the light spot must be less than the radius of the receiver lens r_{lens}, which is 9.2mm (PAF2A-18C, Thorlabs) in our manuscript. The offset reaches its maximum value at maximum $r_0 = \sin(60°) \cdot R(d_0, \lambda_0)$.Thus:

$$L \le \frac{r_{lens}}{\tan\left(\theta(r_0,\lambda) - \theta_0(r_0,\lambda_0)\right)} . \tag{S14}$$

For the wavelength of 1539.77nm and 1560.61nm the maximum L that ensure sufficient coupled energy are respectively 0.80m and 0.76m. For the wavelength of 1546.92nm and 1552.52nm the maximum L are respectively 3.23m and 2.66m, which meets the requirements of most indoor optical wireless communication systems.

Hence, for short-range communication, the boundary wavelength is specified as 1539.77nm and 1560.61nm to augment the number of wavelengths multiplexed in the OWC system to elevate high communication speed. While, for long-distance communication, the boundary wavelength is set at 1546.92nm and 1552.52nm to ensure the coupling efficiency of the receiver collimator and the communication performances of the OWC system.

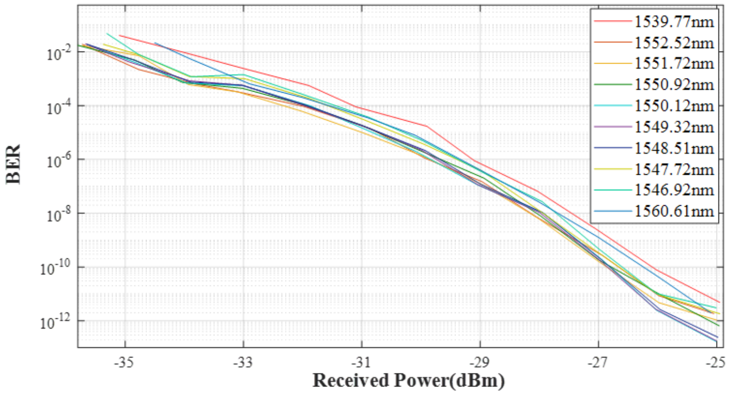

Supplementary Figure S5 | The measured BER-ORP (bit-error-ratio versus optical received power) curves for ten spaced wavelengths from 1539.77 to 1560.61 nm that carried 25Gbits signals.

Furthermore, we have supplemented 1539.77 nm and 1560.61 nm as the carrier wavelengths to the WDM communication characterization. Figure S5 shows the measured bit-error-ratio (BER) curves versus the optical received power (ORP) for the ten wavelengths (specifically: 1539.77nm, 1546.92 nm, 1547.72 nm, 1548.51 nm, 1549.32 nm, 1550.12 nm, 1550.92 nm, 1551.72 nm, and 1552.52 nm, 1560.61nm) at a deflection angle of 30° and a receive distance of 50 cm. The curve indicate that the system could maintain good communication performances when these ten wavelengths are multiplexed. Thus, boundary wavelengths used in WDM communication can be set as 1539.77 nm and 1560.61 nm. It is necessary to clarify that, due to the limitations of the

experimental conditions, only ten wavelengths were multiplexed. However, in practical applications, the number of wavelengths for reuse can be increased to 27 (from C21 1560.61nm to C47 1539.77nm) to ensure high-speed communication over short distances.

Note S4 PRBS signals at different wavelength channels

The FPGA KCU116 Xilinx evaluation board employed in experimental setup hosts four zSFP (z small form-factor pluggable) module connections. Thus, the board features four transceivers for driving the modules as shown in Fig. S6(a). To drive the eight wavelengths optical modules, two FPGA boards with independent clock were used in our work. Due to the asynchronism of different boards, the PRBS sequences generated by different boards are uncorrelated. Moreover, for each FPGA board, the pattern generator blocks of each transceiver can be controlled independently to generate PRBS sequence, as shown in Fig. S6(b). The resets of pattern generators are all asynchronous. Thus, there would be timing differences among the PRBS sequences generated by the same board. As a result, all of the eight PRBS sequences are uncorrelated.

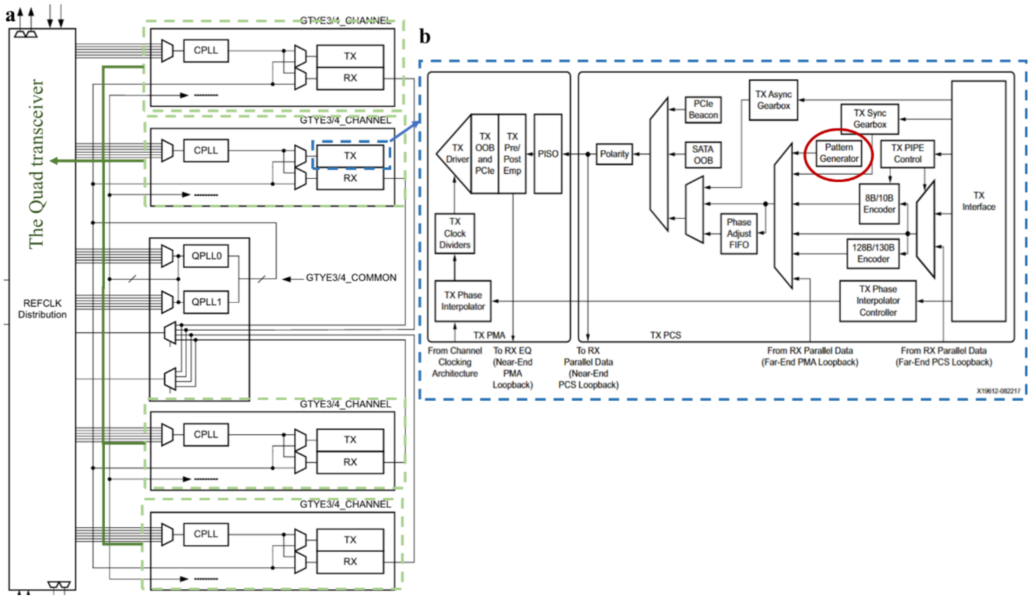

Supplementary Figure S6 | Configuration of the FPGA board. (a) The configuration of Quad transceiver on FPGA KCU116 Xilinx evaluation board, each transceiver is outlined by green dashed lines. **(b)** The block diagram of each transmitter (TX), the pattern generator block is circled in red.

The overlapping tails of different PRBS signals may contribute to the deterioration of BER. However, due to the implementation of DWDM modules (dense wavelength division multiplexing, DWDM-SFP25G-10, Cisco) and the wavelength division multiplexer/demultiplexer (MUX/DMUX, FMU-D402160M3, FS), the interference from adjacent channels is limited. To illustrate the interference between adjacent channels, three PRBS-31 signals carried by wavelengths of λ_1=1549.32 nm, λ_2=1550.12 nm, and λ_3=1550.92 nm were combined by the wavelength division MUX and then demultiplexed by the wavelength division DMUX. the spectral profiles of the optical signal output from the optical module (Fig. S7(a)), wavelength division MUX (Fig. S7(b)) and the wavelength division DMUX (Fig. S7(b)) were recorded by the

spectrometer (AQ6375, YOKOGAWA), as shown in Fig. S7. In Fig. S7(a), the peak of signal spectrum and the lower and upper bounds of the wavelength division channel are demarcated by brown dashed lines. The bandwidth of the DWDM channel is constrained within 0.8 nm as shown in Fig. S7(c). It can be seen from Fig. S7(a) that, the DWDM optical modules possess narrow spectral width. Thus, even though the tail of adjacent channel slight overlaps to the channel bandwidth of the signal, the signal-to-interference ratio (SIR) of adjacent channels still exceeds 40 dB. Therefore, the impact of the adjacent channel interference caused by the overlapping tails to bit error rate (BER) is limited. In summary, for different PRBS signals, although there will be overlap in the tails of adjacent WDM channels, the impact of adjacent channel interference is minimal, allowing for the relatively satisfactory bit error rate (BER).

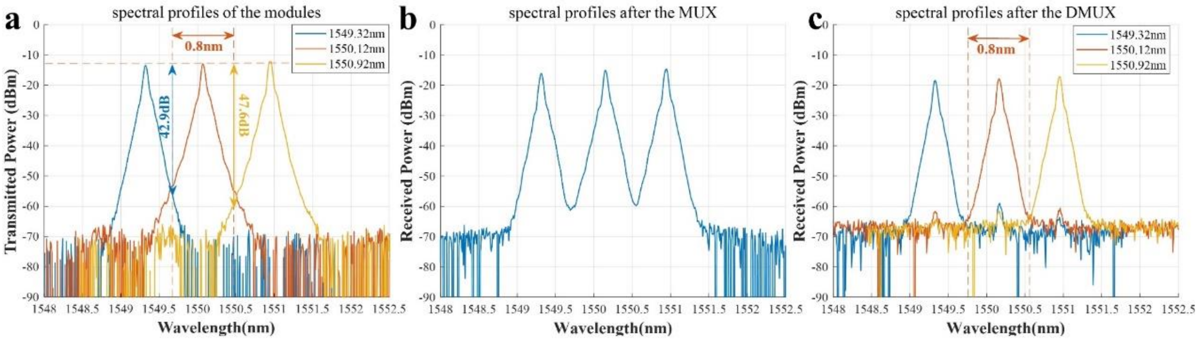

Supplementary Figure S7 | The spectral profiles of the optical signal output, from **(a)** the optical module, **(b)** wavelength division MUX and **(c)** the wavelength division DMUX.

Note S5 Optical power loss of the high-speed wide-field parallel OWC system

In this system as illustrated in Fig. 4, two independent optical links were established for the transmission of optical signals.In optical link 1, video signal displayed by the PC1 was transmitted to a HDMI to SDI converter via an HDMI cable. The signal was subsequently converted to SDI format and transmitted to the Module Compliance Board (MCB, DEB2-12M-A, DooDooTech), which derived an optical module (C38 DWDM-SFP25G-10, Cisco) and transformed the electrical signal into optical signal. The optical signal was coupled into a single mode fiber and emitted into free space after the modulation of the metasurface. After propagating for approximately 1.1m at an angle of about 50°, the signal was coupled into a multimode fiber through a receiver collimator (PAF2A-18C, Thorlabs). The receiving optical signal was then transformed back into HDMI form electrical signal after the MCB and the SDI to HDMI converter. The optical power of the optical module was 1.32 dBm and the received power after the collimator was -10.69 dBm, which falls within the detectable range of the photodiode, enabling direct communication without the necessity of employing transmitter and receiver amplifiers. the total loss of the whole links is about 12dB and includes 2 parts. The first part is a -3.1dB power loss caused by the transmission loss and deflection loss of the metasurface. The second part is the approximately 10dB coupling loss of the receiver collimator.

In optical link 2, dense wavelength division demultiplexing (DWDM) technology was used to improve the capacity. Six spaced wavelengths carried signals were combined by a wavelength division multiplexer (MUX, FMU-D402160M3, FS) and amplified by a EDFA (AEDFA-33-B-FA, Amonics) in order to pre-compensate for the power loss incurred by the MUX, DMUX and the collimator. After modulated by the metasurface and propagating in free space at a distance of 1.2m and the deflection angle of about 25°, the optical signals were coupled into a single-mode fiber by a collimator (PAF2A-18C, Thorlabs) and demultiplexed by the wavelength division demultiplexer (DMUX, FMU-D402160M3, FS). The whole system will cause the optical power loss of about 30dB and includes 3 parts. The first part is the -1.7dB power loss caused by the transmission loss and deflection loss of the metasurface. The second part is the approximately 20dB coupling loss of the receiver collimator to the single mode fiber. The third part are the 4dB insertion loss for each of the MUX and DMUX). It is noteworthy that multimode fiber exhibit enhanced coupling efficiency owing to its relatively wider mode field diameter and larger light harvesting angle in comparison to single mode fiber. Nevertheless, in order to ensure compatibility between the DUMX and the optical module, a single-mode fiber was employed after the collimator. Consequently, the amplifier was incorporated to compensate the signal loss incurred during this process. Furthermore, the coupling loss of the single-mode fibers can also be enhanced by utilizing adjustable fiber coupling kits.

Note S6 Wide coverage achieved by defocusing techniques

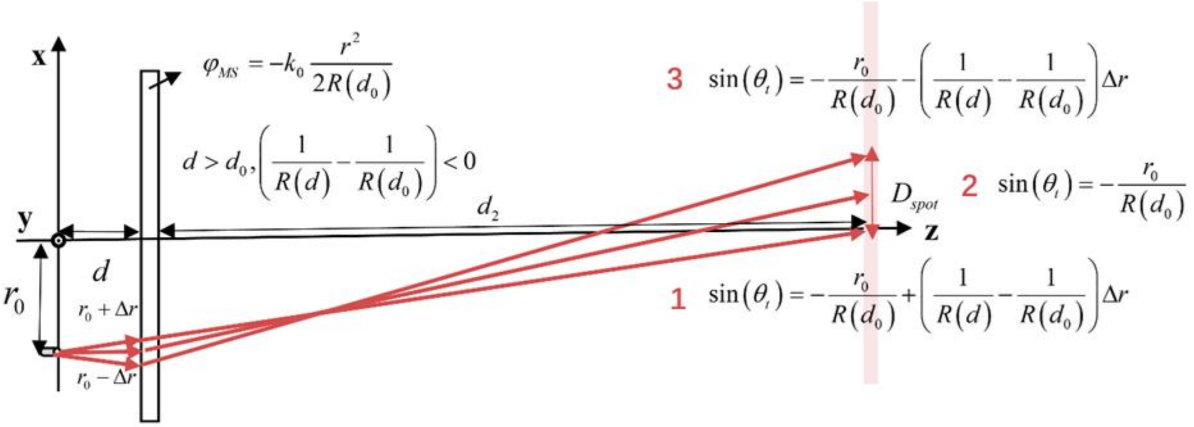

Supplementary Figure S8 | Schematic diagram of defocused beam.

Assuming r_0 is the relative position of the fiber centre to the metasurface centre, d is the distance between the fiber facet and the metasurface. Deflection angle of the light rays at the centre $r = r_0$ and the boundary $r = r_0 \pm \Delta r$ can be expressed as:

$$\begin{cases} k_0 \cdot \sin(\theta_{t1}) = k_0 \cdot \sin(\theta_{i1}) + \left.\frac{\partial \varphi_{MS}}{\partial r}\right|_{r=r_0-\Delta r} = -\frac{r_0}{R(d_0)} + \left(\frac{1}{R(d)} - \frac{1}{R(d_0)}\right)\Delta r \\ k_0 \cdot \sin(\theta_{t2}) = k_0 \cdot \sin(\theta_{i1}) + \left.\frac{\partial \varphi_{MS}}{\partial r}\right|_{r=r_0} = -\frac{r_0}{R(d_0)} \\ k_0 \cdot \sin(\theta_{t2}) = k_0 \cdot \sin(\theta_{i2}) + \left.\frac{\partial \varphi_{MS}}{\partial r}\right|_{r=r_0+\Delta r} = -\frac{r_0}{R(d_0)} - \left(\frac{1}{R(d)} - \frac{1}{R(d_0)}\right)\Delta r \end{cases} \tag{S15}$$

The radial width of the emitted spot can be written as:

$$\begin{aligned} D_{spot,r} &= \left|d_2 \cdot \left(\tan(\theta_{t\max}) - \tan(\theta_{t\min})\right)\right| \\ &= d_2 \cdot \left|\tan\left(\sin^{-1}\left(\frac{-r_0}{R(d_0)} - \left(\frac{\Delta r}{R(d)} - \frac{\Delta r}{R(d_0)}\right)\right)\right) - \tan\left(\sin^{-1}\left(\frac{-r_0}{R(d_0)} + \left(\frac{\Delta r}{R(d)} - \frac{\Delta r}{R(d_0)}\right)\right)\right)\right| \end{aligned} \tag{S16}$$

Since the tangential gradient of the metasurface phase profile is zero, the tangential width of the emitted spot can be approximated as:

$$
\begin{aligned}
D_{spot,t} &= d_2 \cdot \left|\tan\left(\theta_{t\max}\right) - \tan\left(\theta_{t\min}\right)\right| \\
&= d_2 \cdot \left|\tan\left(\sin^{-1}\left(-\left(\frac{1}{R(d)} - \frac{1}{R(d_0)}\right)\cdot \Delta r\right)\right) - \tan\left(\sin^{-1}\left(+\left(\frac{1}{R(d)} - \frac{1}{R(d_0)}\right)\cdot \Delta r\right)\right)\right| \\
&= 2d_2 \cdot \left|\tan\left(\sin^{-1}\left(\left(\frac{1}{R(d)} - \frac{1}{R(d_0)}\right)\cdot \Delta r\right)\right)\right|
\end{aligned}
\quad \text{(S17)}
$$

Therefore, the spot boundary of beam through the metasurface on the plane at the distance of d_2 from the metasurface can be approximated as an ellipse with major axis along $\mathbf{r}$ length $D_{spot,r}$ and minor axis length $D_{spot,t}$, as shown in Fig. S9.

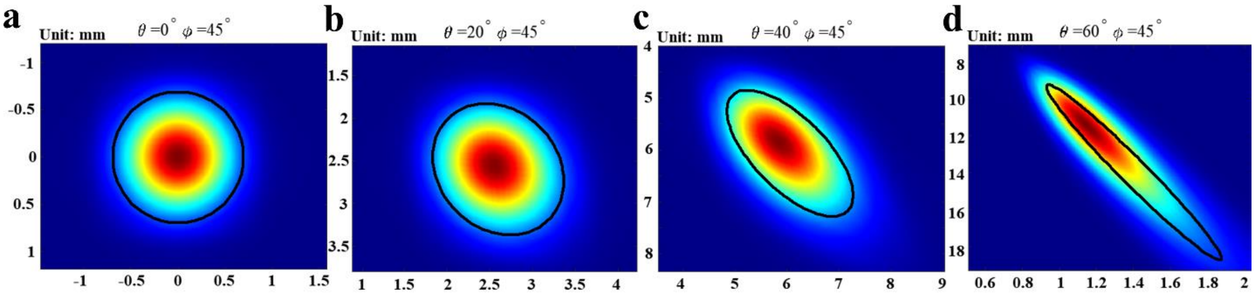

Supplementary Figure S9 | Approximate the defocused beam spot boundaries by elliptic. Comparison of the approximate boundaries and the amplitude distributions calculated by angular spectrum method when $d = 1650\mu m$, $d_2 = 10mm$, $\varphi = 45°$ and **(a)** $\theta = 0°$, **(b)** 20°, **(c)** 40° and **(d)** 60° respectively.

Fig. S9 illustrates the amplitude distributions calculated by angular spectrum method and approximate boundaries of spot expressed by Eq.(S16) and (S17) when $d = 1650.1\mu m$, $d_2 = 10mm$, $\varphi = 45°$, $|\mathbf{r}| = \sin\theta \cdot R(d_0)$ and $\theta = 0°, 20°, 40°, 60°$ respectively. The approximate boundaries are in good agreement with the amplitude distributions, which verifies the rationality of the approximation.

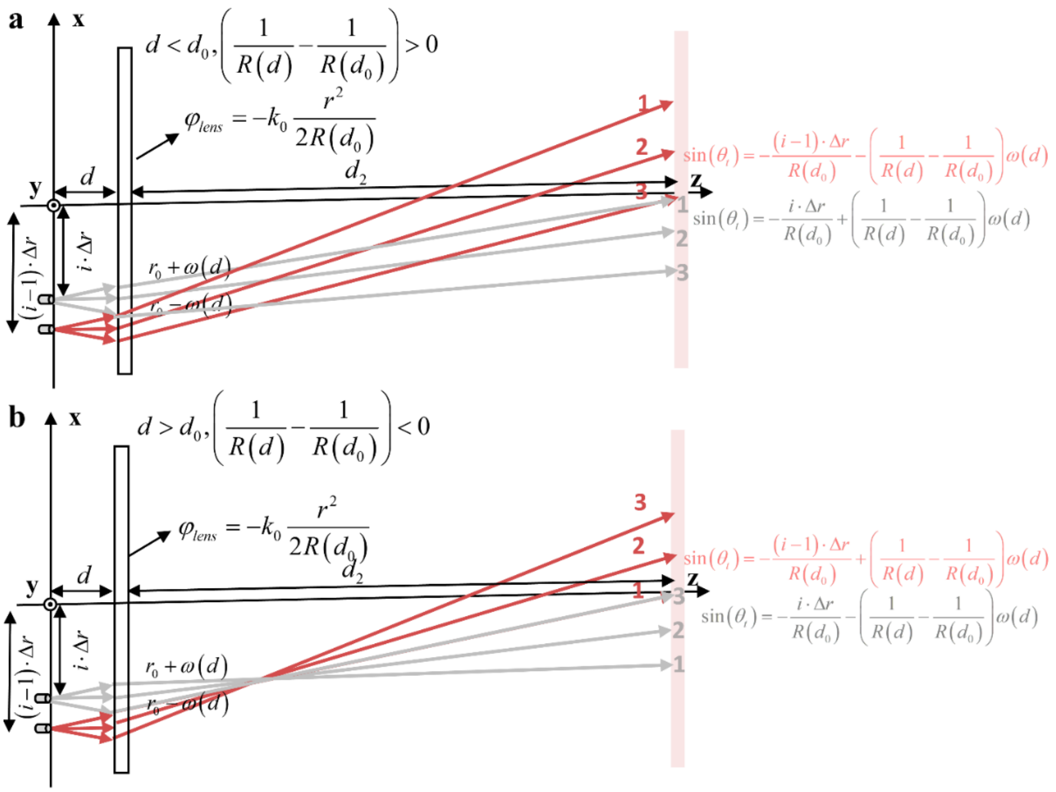

Supplementary Figure S10 | Schematic diagram of full covering. Full coverage achieved by defocused beams when **(a)** $d < d_0$ and **(b)** $d > d_0$.

As shown in Fig. S10, when $d < d_0, \left(1/R(d) - 1/R(d_0)\right) > 0$, the beam through the metasurface exhibits diverging behaviour. Conversely, when $d > d_0, \left(1/R(d) - 1/R(d_0)\right) < 0$, the beam undergoes an initial convergence followed by divergence. To achieve full coverage, it is imperative to maintain consistent deflection angles between adjacent radial beams at the boundary. In other words, for $d < d_0$, the deflection angle of ray 1 from the i th fiber should equal that of ray 3 from the $i-1$th fiber. And for $d > d_0$, the deflection angle of ray 3 from the i th fiber should correspond to that of ray 1 of the $i-1$th fiber. Thus, we get Eq. (S18) for $d < d_0$ and Eq. (S19) for $d > d_0$:

$$\begin{aligned} &\sin(\theta_{ti\max}) = \sin(\theta_{ti-1\min}) \\ &-\frac{i\cdot\Delta r}{R(f)} + \left(\frac{1}{d} - \frac{1}{R(f)}\right)\omega(d) = -\frac{(i-1)\cdot\Delta r}{R(f)} - \left(\frac{1}{d} - \frac{1}{R(f)}\right)\omega(d) \\ &2\cdot\omega(d)\left(\frac{1}{d} - \frac{1}{R(f)}\right) = \frac{\Delta r}{R(f)} \\ &\frac{\left(R(f) - d\right)\cdot\omega(d)}{d} = \frac{\Delta r}{2} \end{aligned} \qquad \text{(S18)}$$

$$
\begin{aligned}
&\sin\left(\theta_{ti\max}\right)=\sin\left(\theta_{ti-1\min}\right)\\
&-\frac{i\cdot\Delta r}{R(f)}-\left(\frac{1}{d}-\frac{1}{R(f)}\right)\omega(d)=-\frac{(i-1)\cdot\Delta r}{R(f)}+\left(\frac{1}{d}-\frac{1}{R(f)}\right)\omega(d)\\
&2\cdot\omega(d)\left(\frac{1}{R(f)}-\frac{1}{d}\right)=\frac{\Delta r}{R(f)}\\
&\frac{\left(d-R(f)\right)\cdot\omega(d)}{d}=\frac{\Delta r}{2}
\end{aligned}
\tag{S19}
$$

To achieve full coverage, the distance between adjacent optical fibers in the radial direction is determined as $\Delta r=\sqrt{2}\cdot p$, submitting $p=100\mu m$ in Eq.(S18) and Eq. (S19), the distance between the metasurface and fiber facet can be obtained as $180\mu m$ and $1650\mu m$. Given the challenges associated with adjusting $180\mu m$, $1650\mu m$ is selected to separate the metasurface and fiber facet. Fig. S11 shows the spatial distribution of amplitude and an approximation of the boundary at $d=1650\mu m$, $d_2=10mm$. As shown in Fig. S11 light beam emitted from the fiber array effectively covers all the designated receiving regions. As a result, their beam spots may significantly overlap at the receiver plane. Leaking energy of other users' carry laser beam that irradiating on the receiving area of this user may cause the crosstalk. For the collimation cases where $d=d_0$, the leaking power from other users is relatively negligible compared to the power of the user's own signal due to the strong directionality and small divergence angle of the carrier laser beam. Nevertheless, when defocusing occurs, the issue of crosstalk becomes significant and cannot be disregarded.

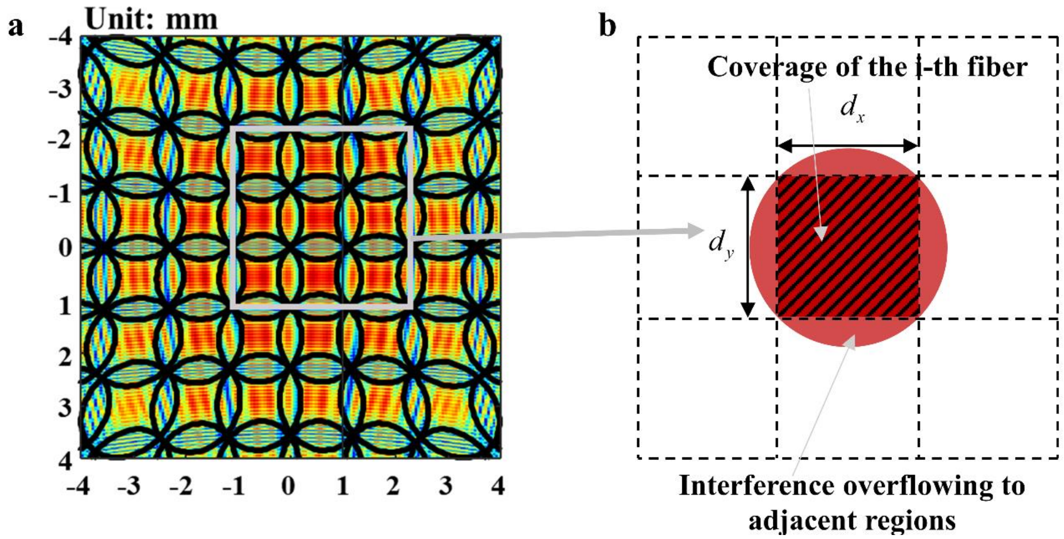

Supplementary Figure S11 | Schematic diagram of full covering and corresponding overlap. **(a)** Spatial distribution of amplitude at $d=1650\mu m$ and $d_2=10mm$, and corresponding

approximated boundary of each beam. **(b)** Schematic diagram of the interfering signals overflow to adjacent regions.

Therefore, to address this concern, the polarization multiplexing technology has been introduced to ensure comprehensive coverage by employing multiple polarizations and simultaneously preventing the overlapping of adjacent light spots within each polarization by multiplexing two orthogonal polarizations. Consequently, interference signals originating from orthogonal polarizations can be theoretically completely eliminated by employing a target polarization filtering approach at the receiving plane. To demonstrate the polarization multiplexed beam-steering metasurface without loss of generality, a multiplexing metasurface with phase profile $\varphi_{LCP} = -k_0\left(x^2+y^2\right)/2R\left(d_0\right)$ for left-circularly polarized (LCP) and phase profile $\varphi_{RCP} = -k_0\left(\left(x-p/2\right)^2+\left(y-p/2\right)^2\right)/2R\left(d_0\right)$ for right-circularly polarized (RCP) is designed. For RCP, when the relative position between the fiber centre and the metasurface centre is $\left(x_0, y_0\right)$, its relative position to the centre of phase profile $\left(p/2, p/2\right)$ is $\left(x_0-p/2, y_0-p/2\right)$, as illustrated in Fig. S12(a). Therefore, when the relative position is $\left(x_0, y_0\right)$, the optical response of one linearly polarized incidence through the polarization multiplexed metasurface is equivalent to that of tow beam located at $\left(x_0, y_0\right)$ and $\left(x_0-p/2, y_0-p/2\right)$ through a metasurface with phase profile of $\varphi = -k_0\cdot\left(x^2+y^2\right)/2R\left(d_0\right)$. In another word, effect of polarization multiplexing can be equivalent to densification the fibers by twice as shown in Fig. S12(b).

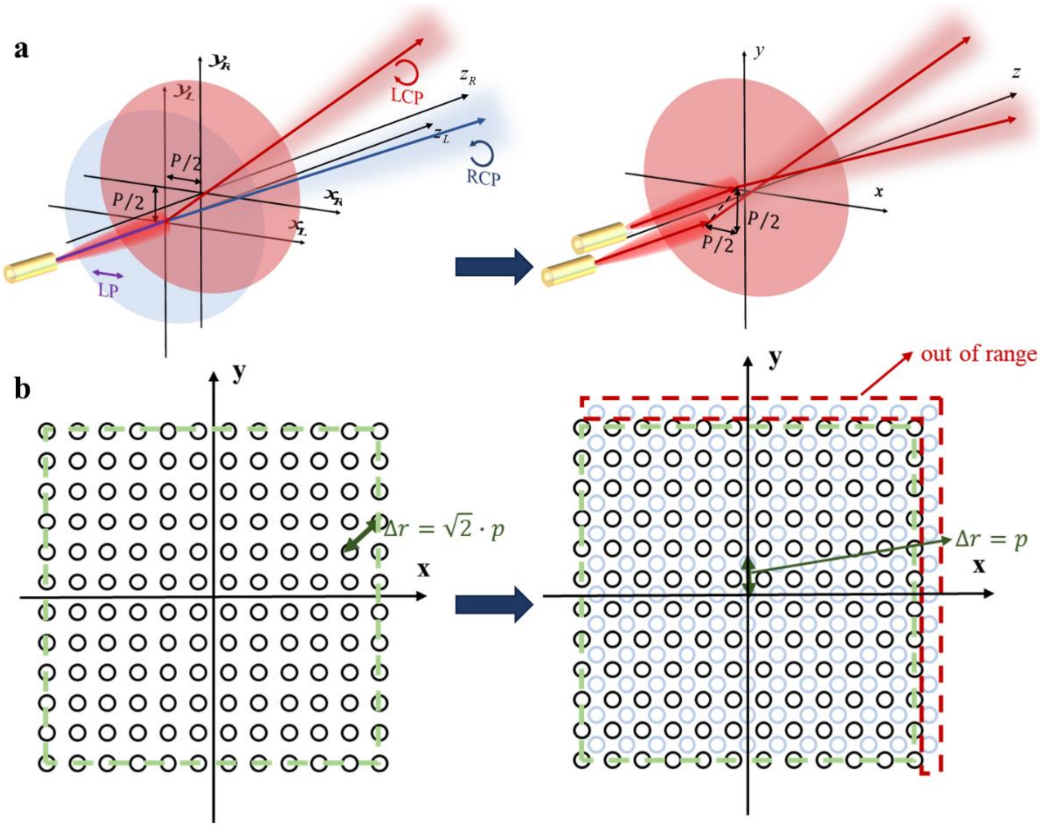

Supplementary Figure S12 | Schematic diagram of crosstalk mitigation utilized by polarisation multiplexing. (a) Schematic diagram of polarization multiplexing metasurface. **(b)** Schematic diagram of equivalent fiber layout.

As shown in Fig. S12(b), the equivalent fiber layout, distance between adjacent fibers in the radial direction is $\Delta r = p$ .submitting $p = 100\mu m$ in Eq. (S19), the distance between the metasurface and fiber facet can be obtained as $1430\mu m$. The amplitude distributions calculated by angular spectrum method and approximated boundaries of spot in the case of full coverage with and without polarization multiplexing are shown in Fig. 5.

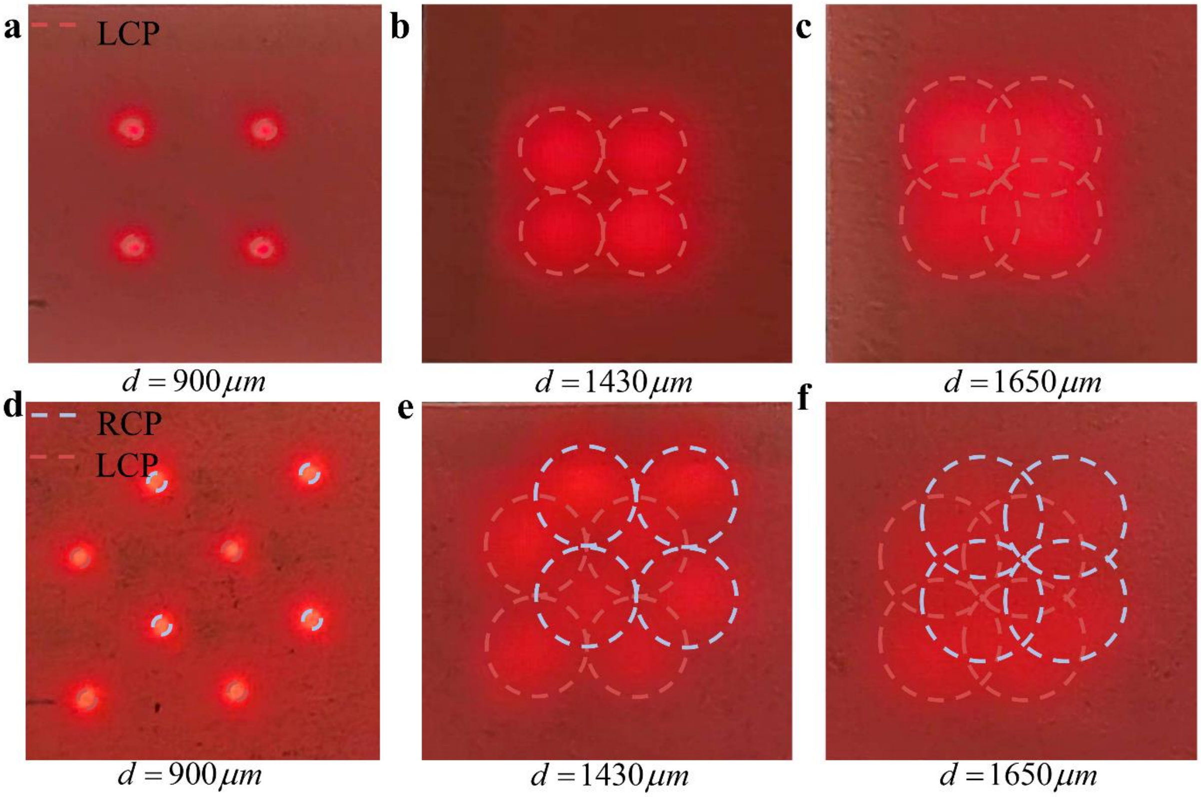

Supplementary Figure S13 | Diffracted beam spot distributions under different incident polarizations. Diffracted beam spot distributions in free space when the distance between the metasurface and fiber array are respectively $900\mu m$, $1430\mu m$ and $1650\mu m$, with **(a-c)** RCP and **(d-f)** LP incidences, when the distance between the IR card and metasurface is about 5cm and the wavelength is 1550 nm.

To demonstrate our concept, the polarization multiplexed metasurface as shown in Fig. 6(b) is fabricated. An IR sensitive card was placed at a distance of about 5 cm after the metasurface sample. Fig. S13 shows the diffracted beam spot distributions in free space when the distance between the metasurface and fiber array are respectively $900\mu m$,$1430\mu m$ and $1650\mu m$, with RCP and linearly polarized (LP) incidences. According to the design in section S8, the polarization of the emitted light demonstrates left circular polarization when the incident light is right circularly polarized. Conversely, the polarization of the emitted light is right circularly polarized when the incident light is left circularly polarized. In the case of LP incidence, the locations of the beam spots represent a combination of LCP and RCP incident beams. Since an LP wave can be regarded as the superposition of LCP and RCP waves with equal amplitudes.

Assuming that all the fibers in the array are active and serving a user, the signal to interference plus noise ratio (SINR) at the user's receiver from the i th fiber is derived as follows:

$$\gamma_i = \frac{R_i^2 \cdot \alpha_i \cdot L_i^2 \cdot h_i^2 \cdot P_i}{\sum_{j \neq i} R_j^2 \cdot \alpha_j \cdot L_j^2 \cdot h_j^2 \cdot P_j + \sigma_i^2} \tag{S20}$$

Where R_i is the receiving efficiency of the receiving, α_i is the attenuation coefficient introduced by the metasurface, L_i is the channel fading from the metasurface to the receiver, P_i is the average power of the symbol, σ_i is the total noise variance. $h_i = \iint_S A_i \cdot dx \cdot dy$ is the ratio of power irradiated in the receiving area to total power, where S_i is the receiving regions and A_i is the amplitude distribution of the beam from i th fiber on the receiving plane. As conditional BER of coherent modulation scheme is $P_e(\tau) = \kappa Q\left(\sqrt{\bar{\gamma}\zeta\tau}\right)$, by improving SIR, γ_i can be improved and P_e can be reduced. Assuming that all users have the same R_i, α_i, L_i, P_i , SIR of user-i can de simplified as $SIR_i = h_i^2 / \sum_{j \neq i} h_j^2$.

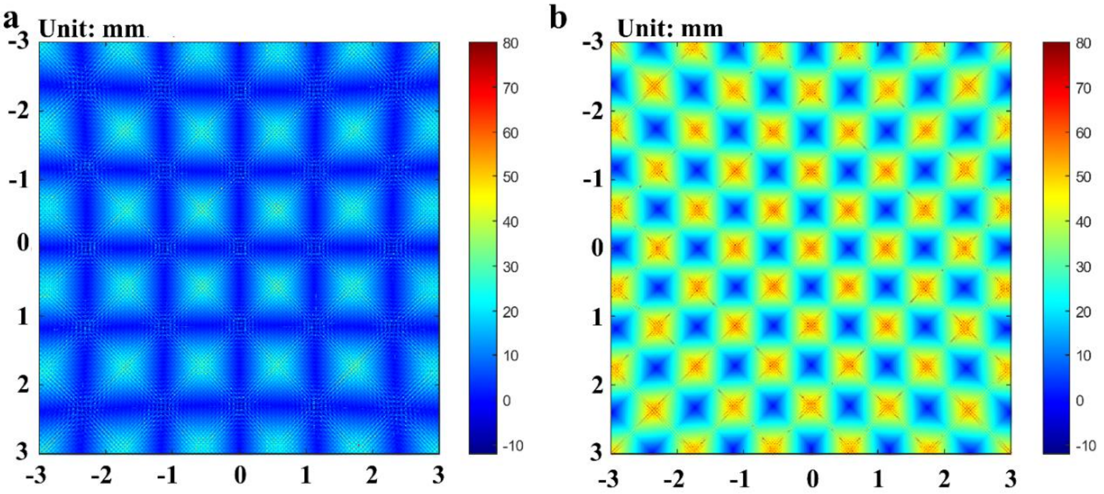

Supplementary Figure S14 | Comparison of received SIR. (a) Spatial distribution of received SIR of the original metasurface at $d = 1650\mu m$ and $d_2 = 10mm$. **(b)** Spatial distribution of received SIR of the polarization multiplexed metasurface at $d = 1430\mu m$ and $d_2 = 10mm$.

Fig. S14 show the spatial distribution of the received SIR at the distance of $d_2 = 10\text{mm}$ where $d = 1650\mu m$ and $d = 1430\mu m$,respectively. As shown in Fig. S14, after introducing polarization multiplexing function, the SIR has been significantly improved. In addition, since P_i from each fiber can be adjusted flexibly, the system performance can also be improved utilizing power allocation schemes.

Note S7 Design of polarization multiplexed metasurface

The beam-steering metasurface is realized by an array of identical resonant consisted of two layers, including a 1μm-thick α-Si film and a SiO_2 substrate. Fig. 6(b) shows a schematic of a unit cell. The unit cell has a period of 800 nm in both the x- and y-direction. Two orthogonal eigen dipoles respectively exist along the short and long axis of such a rectangular nanoantenna. Without rotation (the rotation angle $\alpha = 0$), the Jones matrix of the unit cell can be written as:

$$J = \begin{bmatrix} E_{x0} & 0 \\ 0 & E_{y0} \end{bmatrix} = \begin{bmatrix} \sqrt{T_x} \cdot e^{i\phi_x} & 0 \\ 0 & \sqrt{T_y} \cdot e^{i\phi_y} \end{bmatrix}. \quad \text{(S21)}$$

In Eq. (S21), E_{x0} and E_{x0} represent the complex fields of the two dipoles, where T_x and T_y are the transmittance, φ_x and φ_y are the phase retardation. The complex fields can be controlled by changing the width W and length L of the rectangular nanoantenna. In order to adapt to existing optical communication systems, 1550 nm was chosen as the working wavelength. The phase and transmittance of unit cells with different size at are working wavelength are simulated with the finite-difference time-domain method via commercial software from Lumerical Inc., FDTD Solutions. Periodic boundary conditions are applied along the x and y axis, and perfect matching layers are employed along the z-axis to absorb the outgoing waves.

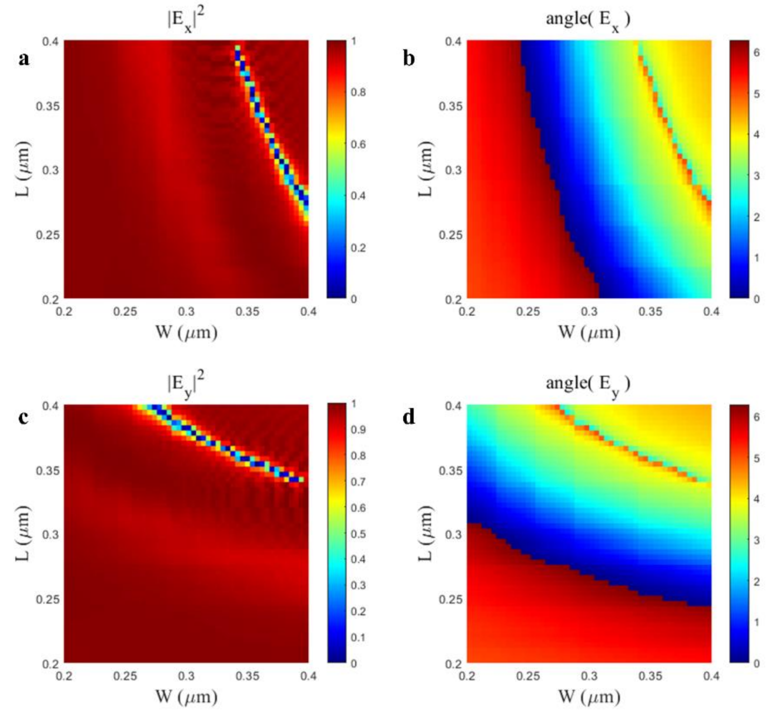

Supplementary Figure S15 | Optical response of identical unit cell. (a) Simulated transmittance and **(b)** phase shift of an identical resonator under x-polarization. **(c)** Simulated transmittance and **(d)** phase shift of an identical resonator under y-polarization.

As depicted in Figure S15, the phase spans an approximate range of 2π, and the transmittance approaches 1 at working wavelength. The Jones matrix of the unit cell with the nano-bar rotate by the angle α is given by:

$$J = R(-\alpha)\cdot\begin{bmatrix} E_{x0} & 0 \\ 0 & E_{y0} \end{bmatrix}\cdot R(\alpha) = \begin{bmatrix} \cos\alpha & -\sin\alpha \\ \sin\alpha & \cos\alpha \end{bmatrix}\cdot\begin{bmatrix} E_{x0} & 0 \\ 0 & E_{y0} \end{bmatrix}\cdot\begin{bmatrix} \cos\alpha & \sin\alpha \\ -\sin\alpha & \cos\alpha \end{bmatrix}, \quad \text{(S22)}$$

where $R(\alpha)$ is the rotation matrix of α , Eq. (S22) can be simplified as:

$$J = \begin{bmatrix} E_x & E_{xy} \\ E_{xy} & E_y \end{bmatrix} = \begin{bmatrix} \cos^2\alpha\cdot E_{x0} + \sin^2\alpha\cdot E_{y0} & \cos\alpha\cdot\sin\alpha\cdot\left(E_{x0} - E_{y0}\right) \\ \cos\alpha\cdot\sin\alpha\cdot\left(E_{x0} - E_{y0}\right) & \sin^2\alpha\cdot E_{x0} + \cos^2\alpha\cdot E_{y0} \end{bmatrix}. \quad \text{(S23)}$$

Assuming that the orthogonal polarization of the incidence and output of the metasurface can be expressed as $\vec{\chi}^+ = \begin{bmatrix} \chi_1^+ & \chi_2^+ \end{bmatrix}^T$, $\vec{\chi}^- = \begin{bmatrix} \chi_1^- & \chi_2^- \end{bmatrix}^T$ and $\vec{\kappa}^+ = \begin{bmatrix} \kappa_1^+ & \kappa_2^+ \end{bmatrix}^T = \left(\vec{\chi}^+\right)^*$ $\vec{\kappa}^- = \begin{bmatrix} \kappa_1^- & \kappa_2^- \end{bmatrix}^T = \left(\vec{\chi}^-\right)^*$ respectively. The optical response of individual unit cells can be expressed as:

$$\begin{aligned} J\cdot\vec{\chi}^+ &= e^{i\varphi^+}\left(\vec{\chi}^+\right)^* \\ J\cdot\vec{\chi}^- &= e^{i\varphi^-}\left(\vec{\chi}^-\right)^* \end{aligned} \quad \text{(S24)}$$

Where φ^+ and φ^- are corresponding phase shifts assigned to the two polarization states. Eq. (S24) can be written as:

$$J\begin{bmatrix} \chi_1^+ & \chi_1^- \\ \chi_2^+ & \chi_2^- \end{bmatrix} = \begin{bmatrix} e^{i\varphi^+}\left(\chi_1^+\right)^* & e^{i\varphi^-}\left(\chi_1^-\right)^* \\ e^{i\varphi^+}\left(\chi_2^+\right)^* & e^{i\varphi^-}\left(\chi_2^-\right)^* \end{bmatrix}. \quad \text{(S25)}$$

Thus, to achieve polarization multiplexing, the Jones matrix of target unit cell needs to meet the requirements of Eq. (S25).

$$J = \begin{bmatrix} e^{i\varphi^+}\left(\chi_1^+\right)^* & e^{i\varphi^-}\left(\chi_1^-\right)^* \\ e^{i\varphi^+}\left(\chi_2^+\right)^* & e^{i\varphi^-}\left(\chi_2^-\right)^* \end{bmatrix}\cdot\begin{bmatrix} \chi_1^+ & \chi_1^- \\ \chi_2^+ & \chi_2^- \end{bmatrix}^{-1}. \quad \text{(S26)}$$

By solving Eq. (S26) the Jones components of the unit cell can be theoretically derived. The period of unit cell is 800nm, by performing the above operations on each unit cell of the metasurface, the Jones matrix of each unit is determined. In this paper a multiplexed metasurface under LCP and RCP with phase profile as shown in Eq. (S27) is designed.

$$\begin{cases} \varphi_{LCP}(x,y) = -k_0\left(x^2+y^2\right)\big/2R(d_0) \\ \varphi_{RCP}(x,y) = -k_0\left[\left(x-p/2\right)^2+\left(y-p/2\right)^2\right]\big/2R(d_0) \end{cases} \quad \text{(S27)}$$

Submitting $\vec{\chi}_{LCP} = \frac{1}{\sqrt{2}}\begin{bmatrix} 1 & -i \end{bmatrix}^T$ and $\vec{\chi}_{RCP} = \frac{1}{\sqrt{2}}\begin{bmatrix} 1 & i \end{bmatrix}^T$ into Eq. (S26), we obtain:

$$J(x,y) = \begin{bmatrix} e^{i\varphi_{LCP}(x,y)} & e^{i\varphi_{RCP}(\mathrm{x,y})} \\ ie^{i\varphi_{LCP}(\mathrm{x,y})} & -ie^{i\varphi_{RCP}(\mathrm{x,y})} \end{bmatrix} \cdot \begin{bmatrix} 1 & 1 \\ -i & i \end{bmatrix}^{-1} = \frac{1}{2}\begin{bmatrix} e^{i\varphi_{LCP}(x,y)} & e^{i\varphi_{RCP}(\mathrm{x,y})} \\ ie^{i\varphi_{LCP}(\mathrm{x,y})} & -ie^{i\varphi_{RCP}(\mathrm{x,y})} \end{bmatrix} \cdot \begin{bmatrix} 1 & i \\ 1 & -i \end{bmatrix}, \quad \text{(S28)}$$

where

$$J(x,y) = R(-\theta) \cdot \begin{bmatrix} E_{x0} & 0 \\ 0 & E_{y0} \end{bmatrix} \cdot R(\theta). \quad \text{(S29)}$$

In Eq. (S29), since $R(\theta) = R(-\theta)^{-1}$, Eq. (S29) can be written as:

$$J(x,y) \cdot R(-\theta) = \begin{bmatrix} E_{x0} & 0 \\ 0 & E_{y0} \end{bmatrix} \cdot R(-\theta). \quad \text{(S30)}$$

Thus, E_{x0} and E_{y0} are two eigenvalues of matrices and the rotation matrix $R(-\alpha)$ is the corresponding eigenvector matrix. The rotation angle α is determined by $R(-\alpha)$. Subsequently, appropriate structural parameters (L, and W) are chosen from the database to satisfy the desired values of E_{x0} and E_{y0}. Given the discretization of L, and W, the structures within the calculated database may not precisely achieve the prescribed E_{x0} and E_{y0}. Consequently, the selection criterion for the structure is to minimize the discrepancy between the E_{x0} and E_{y0} values of the chosen unit cell and the desired E_{x0} and E_{y0} values. This criterion can be formulated as follows:

$$\min\left(\max\left\{\varepsilon_x, \varepsilon_y\right\}\right), \quad \text{(S31)}$$

where $\varepsilon_x = E_{x0} - E'_{x0}$, $\varepsilon_y = E_{y0} - E'_{y0}$ represent the difference between target E_{x0} and E_{y0} of the unit structure and E'_{x0} and E'_{y0} obtained from the simulation database.

Note S8 Detailed SNIR performances comparison of non-polarization multiplexed metasurface method and polarization multiplexed metasurface method

A working signal with carrier wavelength of $\lambda_s=1550.12$nm was coupled into the center fiber, and four interference signals with carrier wavelengths of $\lambda_{I1}=1548.51nm$, $\lambda_{I2}=1549.32nm$, $\lambda_{I3}=1550.92nm$, and $\lambda_{I4}=1551.72nm$ were coupled into the four adjacent fibers, respectively. When using a non-polarization multiplexed metasurface to achieve full range coverage of the communication area, the distribution of the working signal beam and interference signal beams is shown in Fig. S16(a). Behind the metasurface, a receiver is positioned at the working signal beam region (marked by yellow circular curves) to capture all the signals and transmit them to a spectrometer for analysis. The distance between the metasurface and the fiber array under this condition is $d_1=1650\mu m$. The received spectrum normalized to working signal is shown in Fig. S16(b), which is the same with the solid bule line in Fig. 6(d), showing a signal to noise plus interference ratio (SNIR) of about 15dB.

When using a polarization multiplexed metasurface to achieve full range coverage, the input working signal and interference signals were all set as linear polarized (LP). In this case, after passing through the metasurface, there would be both left-handed circular polarization (LCP) modulated beams and right-handed circular polarization (RCP) modulated beams, as shown in Fig. S16(c). The distance between the metasurface and the fiber array is $d_1=1430\mu m$. Under this condition, the LCP and RCP beams surrounding the working signal beam region will all interfere with the working signal. Figure S16(d) shows the received spectrum under this condition without the polarization filtering, from which it can be observed that the interference caused by λ_{I3} and λ_{I4} is greater than that caused by λ_{I1} and λ_{I2}. This is mainly because the RCP beams of λ_{I3} and λ_{I4} are closer to the working signal beam region. The SNIR for λ_{I3} and λ_{I4} is about 15dB, while for λ_{I1} and λ_{I2} is about 22dB.

Figure S16(e) shows the beam distribution after LCP polarizer filtering, in which all the RCP beams are effectively filtered. Under this condition, only the four LCP beams surrounding the working signal beam region attribute to the interference. This interference situation is somewhat similar to the case in the non-polarization multiplexed metasurface method, as shown in Fig. S16(a). However, in this case, the distance between the four interference signal beams regions and the working signal beam region is greater, resulting in a reduced impact of the interference signals on the working signals. Figure S16(f) shows the received spectrum with the polarizer filtering, exhibiting an SNIR of about 25dB, which is the same with the solid brown line in Fig. 6(d). It is a 10dB SNIR performance improvement compared with that in non-polarization multiplexed metasurface method.

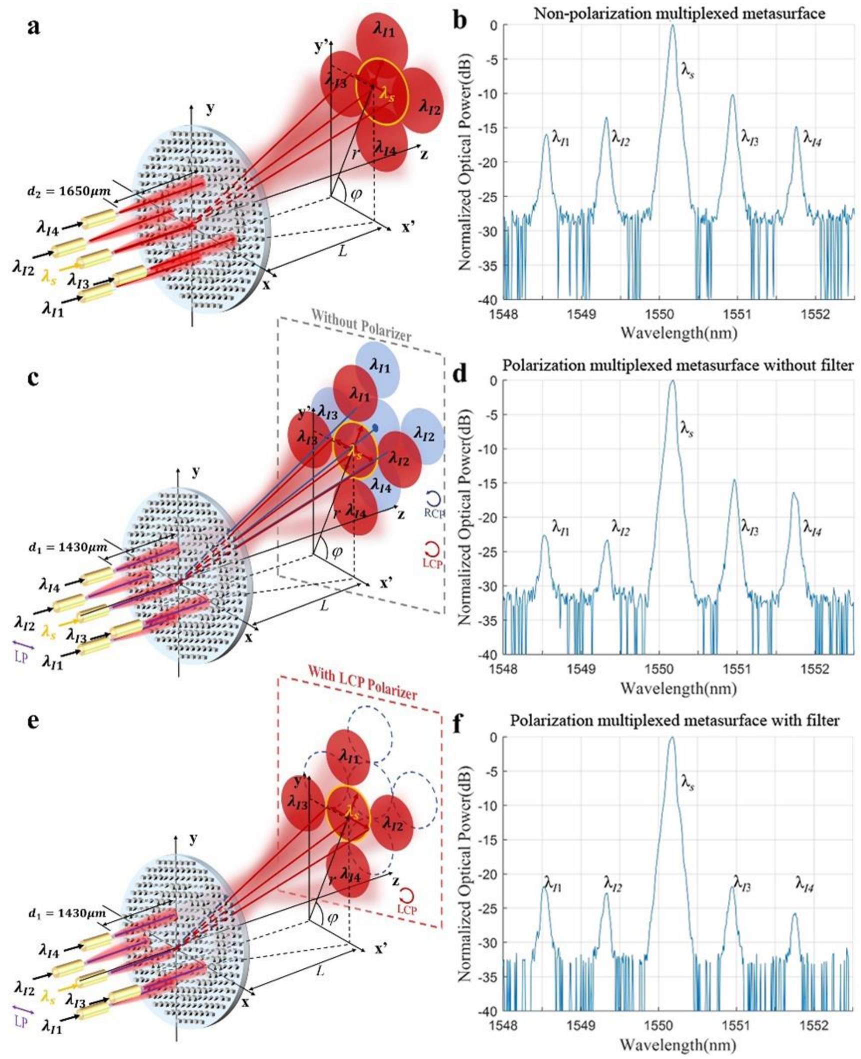

Supplementary Figure S16 | Beam distributions and the corresponding experimental received signals spectrum, of **(a, b)** non-polarization multiplexed metasurface method, **(c, d)** polarization multiplexed method without polarizer filtering, and **(e, f)** polarization multiplexed method with polarizer filtering. The working signal beam regions are marked with yellow circular curves. The red circles in **(c, e)** represent the LCP beam coverage regions, while the blue circles represent the RCP beam coverage regions. λ_S is 1550.12 nm, λ_{I1}-λ_{I4} are 1551.72nm, 1550.92nm, 1549.32nm and 1548.51nm, respectively.

Movie S1.
Movie showing the comprehensive exhibition of the high-speed wide-field parallel OWC system, wherein the communication process is visually represented through animation.

Movie S2.
Movie exhibiting the detailed depiction of signal conversion processes of components in the high-speed wide-field parallel OWC system.

Movie S3.
Movies showing the disconnection and reconnection of the high-speed wide-field parallel OWC system.
