# Supplementary material for: Tbps wide-field parallel optical wireless communications based on a metasurface beam splitter
Source: Nat Commun. 2024 Sep 5;15:7744. doi: 10.1038/s41467-024-52056-4 (PMC11374787; doi:10.1038/s41467-024-52056-4)
Supplement: Supplementary file 3 — Description of Additional Supplementary Files [file 41467_2024_52056_MOESM3_ESM.pdf]

Description of Additional Supplementary Files

File Name: Movie S1
Description: Movie showing the comprehensive exhibition of the high-speed wide-field parallel OWC system, wherein the communication process is visually represented through animation.

File Name: Movie S2
Description: Movie exhibiting the detailed depiction of signal conversion processes of components in the high-speed wide-field parallel OWC system.

File Name: Movie S3
Description: Movies showing the disconnection and reconnection of the high-speed wide-field parallel OWC system.
